# Supplementary material for: Assessment of muscle oxygenation following eccentric exercise-induced muscle damage using near-infrared spectroscopy
Source: PLoS One. 2026 Feb 2;21(2):e0341963. doi: 10.1371/journal.pone.0341963 (PMC12863531; doi:10.1371/journal.pone.0341963)
Supplement: Attachment — Submitted filename: Response to Reviewers.docx [file pone.0341963.s003.docx]

**Reviewer #1**

**Major comments**

**1) L108: Aims of the study: it is not clear why max testing is more convenient than cuff occlusion.**

We thank the reviewer for this helpful comment. Our intention was not to imply that maximal testing is universally “more convenient” than vascular occlusion, but rather that, in applied sport settings, brief maximal or repeated sprint tests can often be implemented more practically than NIRS protocols that require repeated arterial occlusions. To make this rationale clearer, we have revised the final part of the Introduction. We now explicitly (i) describe why occlusion-based NIRS protocols are methodologically demanding for routine applied use and (ii) state that we aimed to evaluate whether a brief, maximal sprint protocol, which aligns with standard performance testing, can provide practically useful NIRS information without the need for vascular occlusion.

Introduction> final two paragraphs

*Current NIRS protocols often rely on vascular occlusion tests or prolonged exercise tasks, which can limit their applicability in applied sport settings (12,13). For example, estimates of muscle oxidative capacity are commonly derived from protocols that combine NIRS with repeated arterial occlusions using pneumatic cuffs inflated to suprasystolic pressures, with multiple inflations and deflations and careful control of blood volume artefacts required to obtain valid estimates (16). In addition, depending on the size and position of the occlusion cuff, these procedures can be uncomfortable or painful, particularly when cuffs are placed proximally on the thigh (21). Such requirements for specialised occlusion systems, trained operators and participant tolerance reduce the feasibility of repeated occlusion-based testing for routine monitoring in team-sport or field environments. In contrast, brief maximal or repeated sprint tests on a cycle ergometer are already embedded within athlete testing and monitoring, have demonstrated reliability and ecological validity, and can be performed using equipment that is commonly available in high-performance settings (22,23). However, it is not yet known whether NIRS indices obtained during such short, high-intensity efforts are sufficiently sensitive to detect changes in muscle oxygenation following EIMD.*

*This study has two primary aims: (1) to determine whether resting TSI in the vastus lateralis is altered following a standardised eccentric squat protocol, and (2) to assess the sensitivity of NIRS-derived variables, including desaturation rate during a sprint and resaturation kinetics during recovery, to EIMD-related changes in muscle oxygenation. By evaluating a protocol that combines resting TSI with a short series of maximal cycle sprints, we intend to test a NIRS approach that can be integrated into existing performance testing without the need for vascular occlusion or prolonged submaximal exercise. Addressing these aims will help to establish the viability of NIRS as a practical tool for monitoring EIMD in applied settings and inform the design of recovery monitoring strategies that are feasible outside of highly controlled laboratory environments.*

**2) L127: why was a moderate reliability (ICC=0.5) used to estimate sample size for control participants? We understand that this allows lowering the required number of participants but it also reduces the strength of the data collected.**

Response combined with (3) below

**3) L135: the same remark also applies to the 80% power used to estimate the sample size of the experimental group.**

We appreciate this comment and agree that the choice of reliability threshold and statistical power should be clearly justified.

For the control group, our primary objective was to characterise day-to-day variability and obtain precise estimates of test–retest reliability (ICC, TE, CV) over six sessions, not to demonstrate very high (e.g. ≥0.90) reliability for applied decision making. We therefore based the a priori calculation on a minimal threshold of interest of ICC = 0.50, using the approach of Walter *et al* for reliability studies. In widely used interpretative guidelines, ICC values <0.50 are considered poor, and values of 0.50–0.75 are considered moderate. Thus, 0.50 marks the lower boundary at which the measure ceases to be regarded as having poor reliability. With six repeated sessions, this threshold yields >99% power with n = 5, which was sufficient for our purpose of quantifying typical day-to-day variation in the absence of EIMD, rather than to make strong claims about excellent reliability.

For the experimental group, the sample size calculation was based on previously published NIRS data following EIMD (Ahmadi *et al.).* From those data, we derived a large effect size (Cohen’s F = 1.39). We then set statistical power at 80%, which is conventionally recommended as a minimum standard in hypothesis-driven research. Given the large effect size and repeated measures design, this yielded a required sample size of 12, which is consistent with previous NIRS studies examining muscle oxygenation responses to eccentric exercise and EIMD that typically include around 8–12 participants.

We fully acknowledge that higher target ICC values and higher power (e.g. 90%) would be desirable in an ideal scenario. However, achieving substantially larger sample sizes is challenging in intensive EIMD protocols involving multiple laboratory visits and detailed physiological measurements. To address the limitations inherent to modest samples, we have emphasised estimation of reliability (ICC, TE, CV) and effect sizes with confidence intervals, rather than relying solely on null-hypothesis significance testing.

To make this rationale explicit, we have revised the Participants section to clarify (i) why ICC = 0.50 was selected as a minimal threshold of interest in the control group, and (ii) why 80% power was adopted for the experimental group, including reference to conventional standards and to sample sizes used in previous NIRS-EIMD work.

Methods>Experimental procedures (Control group)

*In commonly used interpretative guidelines, ICC values below 0.50 are considered to indicate poor reliability, whereas values between 0.50 and 0.75 reflect moderate reliability (25). We therefore selected 0.50 as a minimal threshold to establish that day-to-day variation in the control condition was at least in the moderate range, acknowledging that higher ICC values would be preferable for applied decision-making.*

Methods>Experimental procedures (Experimental group)

*A target power of 80% is widely recommended as a minimum standard for hypothesis-driven studies, balancing the risk of Type II error against the practical constraints of intensive physiological protocols (26). In addition, the resulting sample size is consistent with previous NIRS studies examining muscle oxygenation responses to eccentric exercise and EIMD, which have typically included approximately 9–13 participants (11–13).*

Also, in line with our rationale for selecting ICC = 0.50 as a minimal threshold of interest for the control group, our intention in the Results was to describe the observed reliability indices without overstating the strength of measures that only achieved moderate or poor ICC values. We have therefore revised the reliability paragraph to focus on the numerical ICC and CV values, to use more neutral wording, and to explicitly acknowledge that several NIRS-derived variables showed only moderate or poor reliability despite acceptable within-subject variability. In addition, we have updated the Statistics section to state explicitly that ICC = 0.50 was treated as the minimal threshold at which reliability is no longer considered poor, and to clarify that CV categories are used as descriptive guides rather than strict cut-offs. These changes align the interpretation of the control data and the reporting of reliability metrics with the a priori assumptions used in the sample size calculation and clarify which variables are likely to be most useful for monitoring.

Statistical Analysis section (ICC description)

*In line with commonly used guidelines, an ICC of 0.50 was considered the minimal threshold at which reliability ceases to be regarded as poor, which informed the a priori sample size calculation for the control group.*

Statistical Analysis section (CV description)

*CV thresholds were defined as: <10% (excellent), 10-20% (good), 20-30% (fair), and ≥30% (poor) (38), and are used here as descriptive guides to aid interpretation rather than as strict cut-off criteria.*

Results section

*Table 2 presents the reliability statistics for the NIRS parameters collected from the control participants (n=5), including mean ± SD, CV, TE, SWC, and ICC. Resting TSI report an ICC of 0.91 with a CV of 2.7%, indicating very high within-subject consistency under the standardised laboratory conditions. In contrast, TSI variables derived from the sprint phase demonstrated only moderate reliability (ICC = 0.70) and larger within-subject variability (CV = 20.1%). TSI measures obtained during the recovery phase after Sprint 1 also showed moderate reliability (ICC = 0.64) with a CV of 25.9%, suggesting greater day-to-day fluctuation in these dynamic responses. Time-based variables showed lower ICC values. The duration of the sprint phase had an ICC of 0.44 with a CV of 14.4%, and the duration of the recovery phase after Sprint 1 had an ICC of −0.36 with a CV of 10.4%. These combinations of relatively low CV but poor ICC reflect consistent within-subject measurements alongside notable between-subject differences in these temporal characteristics. Rate of change metrics during the sprint showed an ICC of 0.73 and a CV of 23.9%, whereas recovery rate after Sprint 1 showed an ICC of 0.28 and a CV of 29.1%. Half recovery time yielded an ICC of 0.78 with a CV of 29.3%, indicating that although average values were reasonably consistent at the group level, individual recovery profiles varied between sessions.*

*Reliability statistics for thigh girth, performance, wellness, and CK obtained from control participants during six repeated assessments are shown in Table 3. Mid-thigh girth demonstrated an ICC of 0.99 and a CV of 0.7%, indicating highly consistent anthropometric measurements. The wellness score showed an ICC of 0.88 with a CV of 7.2%, reflecting acceptable consistency in self-reported wellness assessments. Performance measures, including countermovement jump (CMJ) height, mean power, and peak power, all reported ICC values between 0.96 and 0.98 with CVs between 2.8% and 4.2%, confirming high repeatability of these performance outcomes. In contrast, creatine kinase (CK) levels displayed moderate reliability (ICC = 0.59) and higher variability (CV = 35.4%), suggesting significant fluctuations in this biochemical marker between sessions.*

**4) the length of the vertical push is key in jumping performance. Therefore the self-chosen depth used for CMJ assessment is surprising, especially in this kind of setting including repeated measurements over time. How can this methodological issue have impacted on the results? (limitations?)**

We now explicitly acknowledge in the Limitations that allowing self-selected depth may interact with soreness-related changes in joint stiffness and jump strategy, such that participants could subtly adjust countermovement mechanics as EIMD and DOMS developed, potentially masking small changes in neuromuscular function. We also note that future work should consider angle- or displacement-standardised CMJ procedures where tighter control of jump mechanics is required.

Discussion> LImitations

*Second, CMJ depth was self-selected rather than fixed by joint angle or centre-of-mass displacement. Although this approach is consistent with many CMJ monitoring and reliability protocols (43), variation in self-selected depth may have interacted with soreness-related changes in joint stiffness and jump strategy, such that participants subtly adjusted countermovement mechanics as exercise-induced muscle damage and delayed-onset muscle soreness developed, potentially masking small changes in neuromuscular function (44). Future studies could consider angle or displacement standardised CMJ procedures where tighter control of jump mechanics is required.*

**5) L220: how was the linear factor for sprint tests individually determined exactly ?**

We thank the reviewer for raising this point. In revisiting our calculations, we also identified and corrected a reporting error in the original manuscript. We had previously stated that the linear factor was referenced to a cadence of 90 rpm, whereas the calculations were in fact based on a target cadence of 120 rpm.

We have revised the Methods section to (i) give the explicit formula used to calculate the linear factor for each participant, (ii) correct the stated reference cadence from 90 to 120 rev·min⁻¹, and (iii) position our approach in relation to previous linear mode sprint protocols.

Methods>Repeated cycle sprint

*The main protocol comprised ten 6-second maximal effort seated sprints, each separated by a 24-second passive recovery period. During recovery, participants held the pedal cranks in a horizontal position, remained seated, upright and arms fully extended. The ergometer was set in cadence-dependent (linear) mode. The 'linear factor', which governs the flywheel braking resistance as a function of power output/cadence^2^, in which power output is determined by:*

$$\mathrm{Power}\left( W \right)=L \times{cadence}^{2}$$

*Where cadence is expressed in revolutions per minute (rev·min⁻¹) and L is the individual linear factor that sets flywheel resistance. For each participant, the linear factor was individualised using the maximal aerobic power (MAP) obtained during the preceding ramp VO₂max test. A target sprint intensity equal to 300% of MAP at a reference cadence of 120 rev·min⁻¹ was specified, and the linear factor was calculated as:*

$$L = \frac{3 \times MAP}{{120}^{2}}$$

*With this configuration, if a participant attained 120 rev·min⁻¹ during a sprint, the resulting power output would correspond to approximately 300% of their MAP, with higher or lower cadences producing proportionally higher or lower power outputs according to the cadence squared relation. The choice of 120 rev·min⁻¹ as the reference cadence is consistent with evidence that peak cycling power typically occurs at crank velocities around 120 to 160 rev·min⁻¹ (31) and with previous intermittent sprint protocols that set resistance so that supramaximal work rates are achieved at 120 rev·min⁻¹ (32). The 300% MAP target was selected pragmatically, informed by pilot testing and by prior work that has used values of approximately 220–270% of ramp test peak power for repeated 6–30 second sprints on the same ergometer (32).*

**6) the recovery kinetics of tissue saturation index were modelled using a single exponential function. It is not clear how these kinetic parameters were used (not presented) and how the half-recovery time was calculated.**

We thank the reviewer for this helpful comment and agree that our description of the recovery modelling required greater clarity. The monoexponential model was used solely to derive a single summary index, the half-recovery time (t½) of TSI after Sprint 10. The intermediate parameters of the fit (baseline value, asymptote and rate constant) were not used as independent outcome variables, which is why they are not presented separately in the Results.

We have revised the Methods section to explicitly describe this procedure and the calculation of t½.

Methods> NIRS Data Processing:

*TSI was also analysed following the final sprint (Sprint 10) to characterise recovery kinetics. Recovery of TSI after Sprint 10 was modelled in GraphPad Prism (version 10.0.2, Boston, Massachusetts, USA) using a one-phase association monoexponential function:*

Y (*t*)=Y_0_ + (*A*-Y_0_)*(1-exp^(-^*^k*t^*^)^)	(Equation 4)

*Where t is time in seconds from the end of Sprint 10, Y_0_ is the TSI value at the end of the sprint (t = 0), A is the asymptotic recovery value, and k is the rate constant. Data were fitted from the end-sprint value (t = 0 s) to 120 s of passive recovery. From each individual fit, the model-derived half-recovery time (t_1/2_, s) was calculated as:*

$t_{1/2}=\frac{\ln(2)}{k}$	(Equation 5)

*representing the time required for TSI to recover 50% of the change between Y_0_ and A. This half-recovery time was used as the primary kinetic outcome for reliability analysis and to examine the effect of EIMD on recovery of muscle oxygenation.*

**7/ How was mid-thigh skinfold used in NIRS data analysis and interpretation-?**

We agree that subcutaneous adipose tissue thickness (ATT) is an important consideration for NIRS, as it can attenuate the muscle signal and alter absolute saturation values. In our study, mid-thigh skinfold was measured at the NIRS optode site primarily to confirm that ATT was low relative to the source–detector separation, rather than to be used as a covariate or for signal correction.

To make this clearer, we have now (i) described how mid-thigh skinfold was measured and interpreted in relation to the 35 mm optode spacing in the Methods, and (ii) added a brief statement in the Limitations acknowledging that we did not adjust NIRS indices for ATT, so the findings are most applicable to lean, physically active populations with similar low skinfold values.

Methods> Near Infrared Spectroscopy (NIRS)

*Mid-thigh skinfold at the NIRS optode site was measured in triplicate using Harpenden skinfold callipers, and the median value was recorded. Adipose tissue thickness was approximated as half the skinfold value. The NIRS probe used a source–detector separation of 35 mm, which corresponds to an effective sampling depth of ~17–18 mm in muscle.*

Discussion>Limitations

*Finally, although mid-thigh skinfold was low in this sample and within recommended limits relative to the 35 mm inter-optode distance, we did not explicitly adjust NIRS variables for adipose tissue thickness; given that ATT can influence the amplitude of NIRS signals, the present findings are most directly applicable to similarly lean, physically active populations.*

**8) Data used to compute the wellness score should be presented, especially the DOMS score, very relevant in this particular study.**

We thank the reviewer for this suggestion and agree that delayed-onset muscle soreness (DOMS) is conceptually relevant in the context of EIMD. However, we deliberately did not treat the wellness questionnaire as a primary or standalone DOMS instrument in this study.

The wellness tool we used is a brief composite athlete-monitoring measure incorporating sleep quality, fatigue, soreness, stress and mood on a 5-point Likert scale, summed to provide a single global wellness score. This type of tool is commonly implemented as a global indicator of perceived recovery or readiness rather than a domain-specific, validated measure of muscle soreness. It is typically used to provide contextual information alongside primary physiological or performance outcomes, not to diagnose or quantify DOMS per se. Treating the soreness item in isolation as a formal DOMS outcome would therefore be beyond the original purpose of the instrument and not supported by specific validation as a single-item DOMS scale.

In this study, the primary outcome, and the variable on which our sample size was powered, was the change in resting TSI following EIMD. The wellness score was included as an exploratory contextual variable to describe self-perceived status across visits and to support interpretation of the physiological data, not as a primary endpoint. Breaking down the composite score post hoc to foreground a single item as a DOMS outcome would risk over-interpreting a non-validated measure and, in our view, would not strengthen the manuscript.

For these reasons, we have retained wellness as a composite descriptive measure and have not added item-level data or specific DOMS analyses. To avoid any misunderstanding, we have slightly clarified the wording in the Methods to emphasise that the wellness questionnaire was used as a global contextual indicator rather than as a validated DOMS measure.

Methods>Wellness Score

*This brief questionnaire was used as a global indicator of perceived wellness and readiness to train, rather than as a domain-specific validated measure of delayed-onset muscle soreness.*

**9) The extent of EIMD is not easy to evaluate in this study. No significant muscle swelling (mid-thigh girth), no DOMS score provided, no change in peak and mean sprint power and no significant change in CMJ. Effect size for CK difference was moderately to extremely large but with very large interindividual variability. This suggest that some participants might have experienced EIMD while some others did not. Would it be possible to identify subgroups (EIMD vs no EIMD) in your population?**

We agree that indirect markers of EIMD often show heterogeneous responses and that evaluating the overall “extent” of damage is challenging. This complexity is well documented, with substantial interindividual variability in CK, soreness and functional changes after standardised eccentric protocols, and only modest correlations between these markers.

In our experimental group, all participants displayed a clear biochemical response to the EIMD protocol:

1. Mean peak CK increased ~15.7-fold above baseline
2. Individual peak CK responses ranged from ~5.3 fold to ~41.4 fold above baseline (approximately +428% to +4039%),
3. Eight participants peaked at 24 h and four at 48 h post exercise, with none peaking later than 48 h.

Thus, every participant in the EIMD group showed a substantial rise in CK over the 96 h follow-up, consistent with the presence of muscle damage, albeit with different magnitudes and time courses. This pattern aligns with the wider literature, which emphasises very large between-subject variability in CK responses even when exercise is standardised, and cautions against rigid cut-offs for defining “responders” and “non responders”.

We fully acknowledge that other indirect markers, such as mid-thigh girth, CMJ height and sprint power, did not show statistically significant mean decrements. However, dissociations between biochemical, mechanical and perceptual indices of damage are common. Studies frequently report large CK increases with relatively modest or short-lived changes in functional performance, and weak or inconsistent correlations between CK, soreness and strength loss. Our EIMD model, therefore, appears to have induced a moderate degree of muscle damage with a strong biochemical signal and relatively preserved performance, which is consistent with the concept of EIMD as a continuum rather than a binary state.

Regarding the suggestion to create subgroups (EIMD vs no EIMD), we do not consider this feasible or statistically appropriate for this dataset fo the following reasons:

1. Absence of a genuine “no EIMD” subgroup

All experimental participants exhibited substantial CK elevations, and the minimum fold increase (>5× baseline) is well above the typical day-to-day fluctuation.

2. Arbitrary thresholds and small sample size

There is no universally accepted CK cut-off to define the presence or absence of EIMD, and proposed thresholds (e.g. 2–3× baseline, or fixed IU·L⁻¹ values) are context dependent and somewhat arbitrary. With n = 12 in the experimental group, any post hoc median split or cluster analysis based on CK or other markers would be underpowered, statistically unstable and prone to spurious differences.

3. Continuous rather than categorical construct

Contemporary work increasingly treats EIMD as a graded phenomenon, with individuals distributed along a spectrum of damage severity rather than falling into discrete categories. Our primary interest was whether a relatively standardised eccentric protocol that clearly perturbed muscle homeostasis in all participants was sufficient to elicit detectable changes in resting and dynamic NIRS indices, not to explain interindividual susceptibility.

For these reasons, we have not conducted post hoc subgroup analyses and prefer to interpret the NIRS responses in the context of a continuous range of EIMD severity.

**10) Little data are presented from the repeated sprint test (only peak and mean power). Could the authors also include the reduction in peak or mean power from the 1st to the 10th? What about total mechanical work over the whole repeated sprint test?**

We appreciate the interest in a more detailed characterisation of the repeated sprint performance data. In this study, however, the repeated sprint protocol was implemented primarily as a standardised physiological stimulus to provoke desaturation and resaturation in TSI, rather than as a comprehensive repeated-sprint ability test.

Our a priori aims and sample size calculations were centred on NIRS-derived outcomes (resting TSI and recovery kinetics), not on mechanical fatigue indices. We therefore report Sprint 1 peak and mean power to confirm that the initial efforts were genuinely high intensity and to provide contextual information on the workload that may help explain the observed TSI responses during the sprint and subsequent recovery. Beyond this, we did not intend to undertake a full performance analysis of the repeated-sprint protocol.

Derived metrics such as percentage decrement in peak or mean power and total mechanical work are common in repeated-sprint ability research, but they tend to show variable reliability in small samples and would add several exploratory outcomes that are not directly linked to our primary research question. This is particularly the case here, where the half-recovery time of TSI after Sprint 10 did not differ meaningfully across time points; additional performance-derived indices would, in our view, complicate interpretation without altering the main conclusions.

Consistent with this rationale, and as stated in the Methods, although participants completed ten sprints, NIRS analysis was deliberately restricted to three key time points (Sprint 1, recovery after Sprint 1 and recovery after Sprint 10) to balance practicality, signal quality and interpretability. We adopted the same philosophy for the mechanical data, using Sprint 1 peak and mean power to characterise the intensity of the sprint stimulus and to contextualise the NIRS responses, rather than developing a full repeated-sprint performance profile. For these reasons, we have chosen not to add further fatigue metrics (e.g. percentage decrement or total work) to the Results.

**11) Did the authors try to correlate functional parameters (CMJ and sprint tests) with NIRS indices? This could help establish how NIRS-related indices could help monitoring EIMD.**

We agree that the relationship between functional outcomes (e.g. CMJ and sprint performance) and NIRS-derived indices is an important question for future work, particularly from an applied monitoring perspective. However, this study was neither designed nor powered to address that question robustly.

Our a priori aims and sample size calculations focused on detecting changes in NIRS variables (resting TSI and recovery kinetics) following EIMD and on quantifying their test–retest reliability. The functional measures (CMJ, sprint power) were included primarily to characterise the presence and time course of EIMD, not as predictors or outcomes in a planned correlational framework. With a relatively small experimental sample and several potential combinations of NIRS and functional metrics, post hoc correlation analyses would be underpowered and highly vulnerable to chance findings and multiple-comparison inflation. We were therefore concerned that presenting such exploratory correlations could be misleading, especially in the absence of clear a priori hypotheses about specific variable pairs.

**12) Based on the results presented in table 6, the main message is: 1/ the absolute level of muscle deoxygenation and the rate of muscle deoxygenation tends to be reduced 1h after 10x10 barbell squat at 80% 1RM (large effect size), 2) resting muscle oxygenation tends to be is increased 24h post 10x10 barbell squat at 80% 1RM. No oxygenation changes were observed during recovery after the first (absolute or rate of change) or the 10th sprint (half recovery time). Could the authors discuss better the potential mechanisms underlying these observations and how they compare to previous literature in the field ?**

We thank the reviewer for this helpful comment. We agree that the combination of a blunted TSI desaturation response during Sprint 1 at 1h post EIMD and a modest increase in resting TSI at 24 h warrants a clearer mechanistic explanation and closer integration with the existing NIRS–EIMD literature. We have therefore substantially revised the third paragraph of the Discussion to (i) emphasise the high reproducibility of resting TSI and the small but consistent upward shift at 24 h, (ii) highlight the large effect sizes for reduced magnitude and rate of TSI desaturation at 1 h, and (iii) interpret this pattern in the context of prior work showing that eccentric exercise can disturb the matching of oxygen delivery to utilisation, blunt deoxygenation kinetics and impair microvascular function, as well as studies reporting elevated resting muscle StO₂ after eccentric or downhill exercise. In the same paragraph, we now explicitly note that unchanged TSI half-recovery time suggests that the capacity to re-establish saturation during brief passive recovery is relatively preserved, and that EIMD primarily affects oxygenation during contractions rather than reoxygenation once exercise ceases.

Discussion

*Our NIRS data demonstrate that resting TSI is highly reproducible, with low CV and excellent ICCs, and that it showed a small but consistent upward shift (~2%) at 24 hours post EIMD compared with baseline, the only time point at which a potentially meaningful change was observed. In contrast, dynamic NIRS variables during the sprint phase were more variable, yet effect size analyses revealed large changes in specific measures at 1 hour post EIMD, particularly the magnitude and rate of TSI desaturation during a 6-second sprint (Table 6). Taken together, this pattern is consistent with the view that eccentric exercise-induced muscle damage can disturb the balance between oxygen delivery and utilisation in skeletal muscle during contractions. Studies using NIRS and ultrasound have shown that EIMD can slow the onset of muscle deoxygenation, increase the ratio of oxygen delivery to utilisation during severe intensity exercise (11), and impair indices of microvascular reactivity (14), suggesting that damaged muscle extracts less oxygen for a given convective O₂ supply. In this context, the blunted desaturation and slower desaturation rate we observed at 1 hour may reflect a relative increase in O₂ availability for a given metabolic demand, driven by a combination of altered motor unit recruitment, muscle oedema and local vascular dysfunction in the damaged tissue (19,42). Conversely, the small upward shift in resting TSI at 24 hours aligns with previous reports of elevated resting muscle TSI for 24–48 hours or longer after eccentric exercise or downhill walking (12), which have been attributed to reduced muscle oxygen consumption, increased local blood flow, or both (43). Such changes are thought to reflect a combination of reduced oxidative demand in damaged fibres and residual local hyperaemia driven by vasodilatory metabolites and mediators (e.g. nitric oxide, prostaglandins and histamine) and by the inflammatory response to muscle damage (14,44). Within this EIMD* *model, the absence of marked changes in TSI half recovery time suggests that the capacity to re-establish saturation during passive recovery is largely preserved, and that EIMD primarily influences the balance between oxygen delivery and utilisation during contractions rather than the speed of reoxygenation once exercise ceases (12,14).*

To maintain continuity and avoid repetition, we also made minor edits to the subsequent two paragraphs of the Discussion. These changes streamline the comparison with previous occlusion-based and prolonged-exercise studies, remove duplicated mechanistic statements about delivery–utilisation matching and resting TSI, and focus those paragraphs on explaining how differences in EIMD protocols, exercise modality and NIRS measurement methods likely contribute to the divergent findings across studies.

**13) End of 1st paragraph in discussion: “..suggesting that compromised muscle function following eccentric exercise is at least partly underpinned by perturbations in oxygen delivery or utilization”. This sentence is difficult to follow based on your data, because the only functional test that tends to be impaired after eccentric exercise is CMJ, which is not known to be O2 dependent….**

We thank the reviewer for this helpful comment. We agree that in our dataset, the only clear functional impairment was a reduction in CMJ height, which is not primarily oxygen dependent, and that our original wording implied a stronger causal link between CMJ performance and oxygen delivery than is warranted. We have therefore revised the opening paragraph of the Discussion so that CK, wellness and CMJ are explicitly framed as markers confirming the presence of EIMD, and the NIRS variables are described as complementary indices of local muscle oxygenation in damaged muscle, without inferring that perturbations in oxygen delivery directly explain the observed changes in jump performance.

Discussion

*This study primarily aimed to determine whether NIRS–derived indices, measured at rest and during dynamic exercise, are sensitive to potential alterations in muscle oxygenation associated with exercise‐induced muscle damage and recovery status. Although conventional statistical analyses did not reveal significant differences over time or between groups, the magnitude of the observed effect sizes, particularly those associated with elevated creatine kinase, reduced self‐reported wellness, and reduced countermovement jump performance, highlights the practical relevance of these variables as markers of muscle damage and its functional consequences. These markers were included to verify that the eccentric squat protocol induced a meaningful EIMD response, and the NIRS outcomes were then used to characterise any accompanying changes in local oxygenation. We therefore interpret the NIRS findings as describing how oxygen delivery and utilisation behave in a muscle that is known to be damaged, rather than as evidence that impaired oxygen availability directly explains the observed decline in jump performance.*

**14) End of 3rd paragraph in discussion: “… whereby local blood flow to the exercising limb remains elevated during the early recovery period…” What could the mechanisms behind this ?**

We thank the reviewer for this comment. We agree that our original wording (“remains elevated during the early recovery period”) was too vague and insufficiently supported mechanistically. In revising the third paragraph of the Discussion, we have (i) removed that specific phrasing and (ii) reworded the text to frame the small increase in resting TSI at 24 h as being compatible with reduced muscle oxygen consumption and/or residual local hyperaemia, with a brief description of plausible mechanisms.

Discussion

*Conversely, the small upward shift in resting TSI at 24 hours aligns with previous reports of elevated resting muscle TSI for 24–48 hours or longer after eccentric exercise or downhill walking (12), which have been attributed to reduced muscle oxygen consumption, increased local blood flow, or both (43). Such changes are thought to reflect a combination of reduced oxidative demand in damaged fibres and residual local hyperaemia driven by vasodilatory metabolites and mediators (e.g. nitric oxide, prostaglandins and histamine) and by the inflammatory response to muscle damage (14,44). Within this EIMD model, the absence of marked changes in TSI half recovery time suggests that the capacity to re-establish saturation during passive recovery is largely preserved, and that EIMD primarily influences the balance between oxygen delivery and utilisation during contractions rather than the speed of reoxygenation once exercise ceases (12,14).*

**15) Only the first sprint of the repeated sprint test was analysed for deoxygenation and reoxygenation magnitude and kinetics. What about the other sprints? Is only the first sprint informative ? Could the NIRS responses be even more significant later on during the test?**

We agree that, in principle, NIRS responses across all sprints could provide additional information about how muscle oxygenation behaves as fatigue develops. However, this was not the primary focus of the present study.

Our a priori aims and sample size calculations centred on resting TSI and a small set of NIRS indices capturing desaturation and resaturation around a single, clearly defined sprint stimulus, rather than on characterising the full repeated-sprint profile. As outlined in the Methods, we deliberately restricted NIRS analysis to three key segments: (i) Sprint 1, (ii) recovery after Sprint 1, and (iii) recovery after Sprint 10. This design was chosen to balance physiological relevance, practicality and signal quality:

Sprint 1 provides a standardised, “fresh” high-intensity bout with minimal preceding fatigue, allowing us to compare like-for-like desaturation responses across time points and between EIMD and control conditions. Recovery after Sprint 1 and after Sprint 10 were then used to derive a recovery kinetic outcome, giving two anchor points for reoxygenation behaviour without requiring analysis of every recovery interval. Expanding the analysis to include NIRS responses for all ten sprints (and nine intervening recovery periods) would substantially increase the number of derived variables and statistical tests in a modest sample, with a high risk of underpowered findings. It would also amplify the influence of factors such as slight changes in posture and movement artefacts.

We therefore view Sprint 1 (plus the two recovery segments) as strategic time points that are sufficient to address our primary question: whether NIRS-derived indices can detect EIMD-related changes in muscle oxygenation under a practical, field-compatible protocol. We acknowledge that additional information might be gained from analysing all sprints in a larger, specifically powered study focused on repeated-sprint dynamics, but we consider this beyond the scope of the present work. To acknowledge this explicitly, we have also added a statement in the Limitations section noting that future work with larger samples and protocols specifically designed to interrogate repeated-sprint dynamics could extend our approach by modelling NIRS responses across all sprints.

Discussion>Limitations

*Future work with larger samples and protocols specifically designed to interrogate repeated-sprint dynamics could extend the present approach by modelling NIRS responses across all sprints; however, this was beyond the scope and primary aims of the current study.*

**16) End of 5th paragraph: “…yet pronounced alteration in resting muscle oxygenation…” This statement is very strong as resting TSI did not demonstrate any statistically significant changes and only increased by about 3%.**

We agree that our original wording overstated the magnitude of the change in resting TSI, given that the increase was small and not statistically significant. In response, we have removed the phrase “ our streamlined assessment strategy highlighted a delayed yet pronounced alteration in resting muscle oxygenation.” from the Discussion and now describe the 24 h change in resting TSI more cautiously as a small upward shift in a highly reproducible measure.

**17) “…suggest that filtering may enhance the capacity of NIRS to detect physiologically meaningful changes in muscle oxygenation following IEMD.” This statement would require a comparison of NIRS data analysed with vs without filtering.**

We agree with the reviewer that our original wording overstated the implications of our data processing approach. In the current manuscript we used a zero phase shift, low pass Butterworth filter to attenuate high frequency noise and obtain smoother NIRS traces for analysis, but we did not formally compare filtered versus unfiltered data. We have therefore revised the final paragraph of the Discussion to remove the claim that filtering may enhance the capacity of NIRS to detect physiologically meaningful changes. The paragraph now simply notes that filtering was applied to improve signal interpretability and that, in this context, the filtered data yielded stable and physiologically plausible desaturation profiles, without inferring any superiority over unfiltered data

Discussion

*The application of a zero-phase shift, low-pass Butterworth filter was used to attenuate high-frequency noise and produce a smoother signal trajectory during the sprint and recovery epochs. In the present analysis, we did not directly compare filtered with unfiltered data; therefore, we cannot determine whether filtering increased the sensitivity of NIRS outcomes to EIMD. However, the filtered traces provided stable and physiologically plausible desaturation profiles from which effect sizes could be derived, and we consider this approach a reasonable compromise between noise reduction and preservation of the underlying signal when NIRS is used in brief, high-intensity protocols.*

**18) Limitations: how repeated sprint actually impacted on muscle oxygenation responses?**

We understand the reviewer’s concern that the repeated sprint protocol itself could have influenced the muscle oxygenation responses and therefore represents an additional limitation. To make this clearer, we have expanded the Limitations to describe these potential influences on NIRS responses and to note that future studies monitoring recovery over several days may wish to reduce the volume and frequency of maximal effort tests, or separate them from key NIRS assessments, to minimise the risk that the assessment protocol itself delays or alters the underlying recovery trajectory.

Discussion>Limitations

*The repeated sprint protocol employed in this study may have confounded the recovery process. The daily repeated sprints could have introduced cumulative fatigue and additional muscle damage, thereby affecting both recovery markers and NIRS-derived parameters, for example, by altering baseline fatigue state, motor unit recruitment patterns or local perfusion. This repeated exposure might have blurred the distinction between the EIMD and control groups over the testing period, as the recovery process was potentially compromised by cumulative exercise stress (47). Evidence from comparable protocols involving short-duration maximal efforts (8 x 5-second efforts) with active recovery has shown no significant functional impairments 24 hours post-exercise (48). Given the similarity to our 10 × 6-second repeated sprint protocol, this is unlikely to have induced additional muscle stress that influenced the NIRS outcomes, particularly as both groups completed the same sprint work. Future work with larger samples and protocols specifically designed to interrogate repeated-sprint dynamics could extend the present approach by modelling NIRS responses across all sprints; however, this was beyond the scope and primary aims of the current study. In addition, future studies that monitor recovery over several days may wish to minimise the volume and frequency of maximal-effort tests, or to separate them temporally from key NIRS assessments, to reduce the risk that the assessment protocol itself delays or alters the underlying recovery trajectory.*

**19) Conclusion is not fully supported by the present data. For instance: “this study demonstrates that NIRS-derived indices are sensitive to muscle oxygenation perturbations following exercise-induced muscle damage.” NIRS was used to monitor muscle oxygen status, so you observed changes in NIRS-derived indices but to what extent it actually relates to modifications in muscle oxygenation or muscle damage remains unclear. Correlation analyses could help in this regard.**

Response combined with (20) below

**20) Conclusion: “…for monitoring recovery and underscore its potential application in both athletic and clinical settings”. NIRS-derived parameters were not statistically significantly altered post eccentric exercises and only effect-size inferences indicate potential meaningful changes at 1h et 24h post exercise. It is not clear how to interpret these findings in terms of recovery from muscle damage. Does that mean possible muscle damage fully recovered at 48h post eccentric exercise? Potential applications in both athletic and clinical settings are mentioned several times throughout the manuscript but it is not clear how the authors actually see them?**

We appreciate the reviewer’s concerns regarding the strength and interpretation of our conclusions. In light of this, we have revised the Conclusion to adopt more cautious language. The revised text now states that, in this small cohort, resting TSI showed excellent reproducibility and that selected NIRS-derived indices exhibited small but systematic changes in temporal association with an eccentric squat protocol that elicited biochemical, perceptual and functional markers of muscle damage, rather than claiming that the study “demonstrates” sensitivity of NIRS to EIMD. We also no longer infer that these findings establish NIRS as a recovery-monitoring tool. Instead, we emphasise that the observations are preliminary and that larger, hypothesis-driven studies are required to better characterise how NIRS-derived indices relate to the severity and time course of muscle damage and to establish whether, and in what form, such measures can be integrated into applied athletic or clinical monitoring.

Conclusion

*In this cohort of healthy, physically active adults, resting tissue saturation index (TSI) measured with NIRS showed excellent reproducibility across repeated days, and selected NIRS derived indices exhibited small but systematic changes in temporal association with an eccentric squat protocol that elicited biochemical, perceptual and functional markers of exercise-induced muscle damage. Although traditional statistical tests did not identify significant group-by-time effects, effect size analyses indicated a modest upward shift in resting TSI at 24 hours post EIMD and large changes in the magnitude and rate of TSI desaturation during a 6-second sprint at 1 hour. These observations suggest that NIRS measures may have the potential to contribute to non-invasive characterisation of muscle status during recovery from unaccustomed eccentric exercise; however, the present findings should be regarded as preliminary. Larger, hypothesis-driven studies are required to better characterise how NIRS derived indices relate to the severity and time course of muscle damage and to establish whether, and in what form, these measures can be integrated into applied athletic or clinical monitoring.*

**Minor comments**

**1/ L75: “Similarly…” this sentence (acceleration of oxygen desaturation) seems to indicate the opposite than the previous one (slow deoxygenation). Need to adapt ?**

We thank the reviewer for pointing out this inconsistency. We agree that the original wording with “Similarly” was misleading, since the eccentric squat and downhill walking studies report slowed and accelerated deoxygenation, respectively. We have revised the paragraph to remove “Similarly”, to describe downhill walking as a different EIMD model, and to state explicitly that these studies show altered (rather than uniformly slowed) deoxygenation kinetics. The new text also clarifies that, despite opposite directional changes, both models indicate that muscle damage can disturb normal deoxygenation kinetics.

Introduction

*In a different EIMD model, exhaustive downhill walking has been shown to accelerate oxygen desaturation during isometric contractions at 30%, 50%, and 80% MVC, with desaturation and resaturation kinetics remaining altered for up to four days post-exercise (12). These findings, despite their differing directional effects, suggest that muscle damage can disturb normal deoxygenation kinetics and may reflect microvascular dysfunction and increased energy demands for muscle repair (12)*

**2/ L89: these effects are not necessarily observed. This sentence should indicate unaccustomed or high-intensity eccentric exercises can lead to muscle damage**

We agree that not all eccentric contractions necessarily produce these effects. We have therefore revised the sentence to specify that unaccustomed or high-intensity eccentric contractions are particularly disruptive and can lead to focal microtears, oedema and elevated intramuscular pressures, rather than implying that this occurs with all eccentric contractions.

Introduction

*Unaccustomed or high-intensity eccentric contractions are particularly disruptive because the mechanical strain on a reduced number of active sarcomeres can lead to focal microtears, oedema and elevated intramuscular pressures (17,18).*

**3/ L99: “invasive vascular occlusion…” to what methodology do the authors refer to ? Cuff inflation is not invasive**

We agree that cuff-based vascular occlusion tests are not invasive, and that our original wording was misleading. We have therefore revised the sentence to read: “Current NIRS protocols often rely on vascular occlusion tests or prolonged exercise tasks, which can limit their applicability in applied sport settings (12,13).

**4/ L153: why was stretching “optional” ?**

We agree that the original wording was unclear. Light stretching was not intended as part of the experimental protocol; it was simply allowed during the short pause after the warm-up to maximise participant comfort, recognising that some individuals prefer to stretch before a maximal test while others do not. It was not prescribed, standardised or recorded, and was not expected to materially influence VO₂max or MAP. To avoid confusion, we have revised the Methods to clarify that participants were permitted to perform light, self-selected stretching before the incremental test, and that this was for comfort only and not part of the experimental manipulation.

Methods>Maximal incremental cycle test

*Participants performed a 5-minute 50 W warm-up at 80 ± 10 rpm.* ***During the brief pause before the incremental test, they were permitted to perform light, self-selected stretching if desired, but this was not prescribed and was intended solely to maximise comfort rather than to influence test performance.***

**5/ L157: a ref is lacking for VO2max criteria**

We thank the reviewer for noting this. We have now added an appropriate reference to support the VO₂max criteria used in the incremental test (Cunha et al., Int J Sports Med, 2010).

**6/ L164: a 5s time window seems rather short to determine accurate VO2max data. Usually 30s windows are preferred. Could the authors include a ref supporting the 5s time window they used?**

We thank the reviewer for spotting this error. The text incorrectly implied that VO₂max was determined from a 5s time window. In practice, breath-by-breath data were exported as 5s intervals, and a 30s time-averaged value was used to identify VO₂max. We have corrected the Methods accordingly and added a supporting reference on the use of 30 s averaging for VO₂max determination (Midgley et al., 2007, Clin Physiol Funct Imaging).

Methods>Maximal incremental cycle test

*V̇O₂max was defined as the highest 30-s time-averaged V̇O₂ value (28), calculated from breath-by-breath data exported in 5-s intervals.*

**7/ L190: anthropometry**

We thank the reviewer for pointing this out. The term “anthropometry” was misspelt in the original manuscript and has now been corrected.

**8/ L315: “…calculating 0.2 of the between-participant…” Is there a typo error here ?**

We thank the reviewer for this query. There is no typo in this sentence: the smallest worthwhile change (SWC) was intentionally defined as 0.2 × the between-participant standard deviation, consistent with the conventional “small effect” threshold used in sports science and reliability work. To improve clarity, we have rephrased the sentence in the Statistics section.

Statistical Analysis

*The smallest worthwhile change (SWC) was defined as 0.2 times the between-participant standard deviation.*

**9/ L356: “fair variability…” for consistency, wouldn’t it be better to use “moderate variability” ?**

We agree with the reviewer’s suggestion and have revised the wording from “fair variability” to “moderate variability”.

**10/ Figure 4 and figure 5: please indicate the data were collected at sprint 1**

We have now updated the titles of Figures 4 and 5 to state explicitly that the NIRS data were collected during Sprint 1, as requested.

**11/ “highlight practical alterations in oxygen delivery and utilization associated with muscle damage”. It may be more accurate to mention “alterations in O2 delivery/utilization balance”.**

We thank the reviewer for this helpful suggestion. In line with this, we have substantially revised the Conclusion to adopt more cautious and descriptive language. The revised text no longer states that the study “demonstrates” practical alterations in oxygen delivery and utilisation, but instead notes that effect-size analyses indicated a modest upward shift in resting TSI at 24 h and large changes in the magnitude and rate of TSI desaturation at 1 h, and that these observations may reflect alterations in muscle status in temporal association with the EIMD protocol.

**Reviewer #2**

**L 114: could the authors precise what they define by recreationally active male (for instance; training background, weekly training volume, type of activity)? Were all the participants naïve from eccentric solicitations during their physical activities?**

We thank the reviewer for this comment. We have now clarified our use of the term “recreationally active” in the Participants section. Specifically, we state that the sample comprised healthy, non-sedentary young men who regularly engaged in sport or exercise at a social, non-elite level (e.g. university club basketball, football or volleyball, or gym-based resistance and/or aerobic training) on average at least twice per week in the months preceding the study. We also indicate that participants were instructed to avoid strenuous exercise for 72 hours before baseline and to refrain from additional structured exercise or sports participation for the duration of the protocol, and that none had taken part in similar EIMD research protocols in the preceding six months.

Methods>Participants

*Seventeen healthy, recreationally active young men (mean ± SD; age 22 ± 5 years; body mass 76.9 ± 9.4 kg; stature 1.74 ± 0.26 m) were prospectively recruited for this study between 25 April 2014 and 25 May 2014. All participants were non-sedentary and reported regular engagement in sport or exercise at a social, non-elite level (e.g. university club basketball, football or volleyball, or gym-based resistance and/or aerobic training) on average at least twice per week in the months preceding the study. All were free of injury and pre-existing medical conditions, were non-smokers, were not using prescription, anti-inflammatory or analgesic medication and had not been exposed to altitude in the two weeks prior to participation. Participants were instructed to avoid strenuous exercise for 72 hours before baseline testing and to refrain from additional structured exercise or sports participation for the duration of the study, and none had taken part in similar EIMD research protocols in the preceding six months. Ethical approval was obtained from the University of Essex Research Ethics Committee. Written informed consent was provided by all participants prior to inclusion in the study, in accordance with the Declaration of Helsinki.*

**L 120: could the authors report the identification code provided by the UE Research Ethics Committee?**

We thank the reviewer for this comment. The study received institutional ethical approval from the University of Essex Research Ethics Committee, and the approval documentation has been provided as part of the submission. At the time the study was conducted (2014), identification codes were not routinely assigned to these internal applications by the Committee, so no specific ethics reference number is available to report.

**L 126-128: could the authors precise which variable they consider precisely to determine the sample size of the control group (as done for the experimental group)?**

We thank the reviewer for this query. For the control group, the a priori sample size was based on detecting at least moderate test–retest reliability (ICC = 0.50) across six repeated sessions, using the approach of Walter et al. for ICC-based reliability studies, rather than on a specific variable’s variance. This calculation depends on the target ICC, number of repeated measures, α and power, and is not tied to a single outcome measure. Nonetheless, for clarity we have revised the Methods to state that this calculation was applied to our primary NIRS outcomes (resting TSI and sprint-phase TSI indices), and that a sample size of five participants provided >99% power to detect a true ICC of 0.50 in this context.

Methods>Experimental Procedures

*Participants were allocated into either an experimental (EXP) or a control (CON) group. Allocation was sequential rather than randomised. The first five participants were allocated to the CON group to determine the day-to-day variation of the dependent variables. This group did not undergo the EIMD protocol. A priori power analysis was conducted to determine the minimum sample size required to assess within-subject reliability across six repeated measures in a control condition. Targeting a conservative threshold for moderate reliability (ICC = 0.50) for the primary NIRS outcomes (resting TSI and sprint phase TSI indices), it was determined that a sample size of five participants would provide >99% power to detect a true ICC of 0.50 (α = 0.05) (24). In commonly used interpretative guidelines, ICC values below 0.50 are considered to indicate poor reliability, whereas values between 0.50 and 0.75 reflect moderate reliability (25). We therefore selected 0.50 as a minimal threshold to establish that day-to-day variation in the control condition was at least in the moderate range, acknowledging that higher ICC values would be preferable for applied decision-making.*

**L 139: what was done during the familiarization session? Were the participants accustomed to the testing procedure and/ or exercise protocol? Whenever they would familiarize to exercise protocol, how did the experimenter account for induction of neuromuscular fatigue and its recovery, or alternatively, for EIMD?**

We thank the reviewer for raising this point. On the first laboratory visit, participants completed a maximal incremental cycle test, followed by a 30-minute seated rest and then a 1RM barbell squat test. After these maximal assessments, they were familiarised with the remaining procedures and equipment: under investigator supervision, they were shown the NIRS set-up and anthropometric measurements, coached on countermovement jump technique, and performed a small number of practice efforts (approximately three CMJ and three 6s sprints on the cycle ergometer) with ample rest and verbal feedback. No sets from the 10 × 10 eccentric squat protocol were performed during familiarisation, and these practice efforts were not intended to induce neuromuscular fatigue or muscle damage.

As shown in Table 1, this initial visit (incremental test, 1RM and familiarisation) was separated from Visit 2, at which the pre-EIMD baseline measures were collected, by 72 hours to minimise any carry-over of neuromuscular fatigue into the subsequent testing days. We have now clarified this sequencing in the Methods.

Methods>Experimental Procedures

*On the first visit, participants completed a maximal incremental cycle test, followed by a 30-minute seated rest and then a 1-repetition maximum (1RM) barbell squat test. After these maximal assessments, participants were familiarised with the remaining procedures and equipment: under investigator supervision, they were shown the NIRS set-up and anthropometric measurements, coached on countermovement jump technique, and performed a small number of practice efforts (approximately three CMJ and three 6-second sprints on the cycle ergometer) with ample rest and verbal feedback. This sequencing was chosen so that any minor fatigue associated with familiarisation did not carry over into the incremental or 1RM tests performed on that day.*

**L 221: what was the level / intensity of this standardized resistance? Were the participants allowed to stand-up from the saddle when sprinting?**

We thank the reviewer for this comment. In response, we have substantially revised the description of the repeated sprint protocol and the linear factor calculation in the Methods. We now clarify that sprints were performed seated only, and that the ergometer was operated in cadence-dependent linear mode with an individually calculated linear factor set so that, at a reference cadence of 120 rev·min⁻¹, the target power output corresponded to approximately 300% of each participant’s maximal aerobic power (MAP) derived from the incremental test. This provides a supramaximal, but standardised, sprint intensity across participants.

Methods>Repeated cycle sprint

*The main protocol comprised ten 6-second maximal effort seated sprints, each separated by a 24-second passive recovery period. During recovery, participants held the pedal cranks in a horizontal position, remained seated, upright and arms fully extended. The ergometer was set in cadence-dependent (linear) mode. The 'linear factor', which governs the flywheel braking resistance as a function of power output/cadence2, in which power output is determined by:*

$$Power \left( W \right)=L \times{cadence}^{2}$$

*Where cadence is expressed in revolutions per minute (rev·min⁻¹) and L is the individual linear factor that sets flywheel resistance. For each participant, the linear factor was individualised using the maximal aerobic power (MAP) obtained during the preceding ramp VO₂max test. A target sprint intensity equal to 300% of MAP at a reference cadence of 120 rev·min⁻¹ was specified, and the linear factor was calculated as:*

$$L = \frac{3 \times MAP}{{120}^{2}}$$

*With this configuration, if a participant attained 120 rev·min⁻¹ during a sprint, the resulting power output would correspond to approximately 300% of their MAP, with higher or lower cadences producing proportionally higher or lower power outputs according to the cadence squared relation. The choice of 120 rev·min⁻¹ as the reference cadence is consistent with evidence that peak cycling power typically occurs at crank velocities around 120 to 160 rev·min⁻¹ (31) and with previous intermittent sprint protocols that set resistance so that supramaximal work rates are achieved at 120 rev·min⁻¹ (32). The 300% MAP target was selected pragmatically, informed by pilot testing and by prior work that has used values of approximately 220–270% of ramp test peak power for repeated 6–30 second sprints on the same ergometer (32).*

**L 223-224: could the authors precise which power output values were provided by the bicycle ergometer (i.e. maximal, average power over a revolution, other?)? What was the sampling frequency from the Lode?**

We thank the reviewer for this request for clarification. In our set up, the Lode Excalibur Sport ergometer provided a continuous workload signal representing the instantaneous power of the eddy-current brake (the “actual power of the brake” in the manufacturer’s terminology, 1 W resolution). This analogue workload output, together with the cadence output, was fed into a PowerLab 8/30 system and sampled at 10 Hz (10 samples·s⁻¹) using LabChart software. For each 6 s sprint, mean power was calculated as the arithmetic mean of all 10 Hz power samples within the sprint, and peak power was defined as the highest 10 Hz power value observed during that sprint. We have clarified this in the Methods section as shown below.

Methods>Repeated Cycle Sprint

*Pedal cadence and workload (instantaneous mechanical power of the brake) were exported from the ergometer’s analogue outputs to a PowerLab 8/30 system (ADInstruments, Oxford, UK) and sampled at 10 Hz using LabChart 5 Pro software. For each 6-second sprint, mean power was calculated as the average of all 10 Hz power samples within the sprint window, and peak power was defined as the highest 10 Hz power value attained during that sprint.*

**Discussion**

**In my opinion, the first paragraph of the discussion section lacks of relevancy regarding the findings of your study. Specifically, markers of EIMD that you mention are still accepted in the literature to monitor the level of muscular disturbance and performance alteration, so your study only confirm, at most, previous knowledge. That said, I do not understand why these markers are presented at the beginning of this section, while there was no mention of those variables in the objectives or hypothesis.**

**Furthermore, I do not question whether oxygen delivery is impaired following eccentric exercises, but in your study, I am not convinced that reduction in CMJ performance could be explain by reduction in oxygen delivery. Rather, I guess that alteration in CMJ performance would be explain by EIMD and alteration in excitation-contraction coupling process (e.g. calcium release, sensitivity of stretching reflex or perturbations of sarcomeres’ integrity) that would hinder maximal force production capacity.**

**I suggest the authors to reconsider the element discussed in this first paragraph to comply with there main objectives and the state of their findings.**

We thank the reviewer for these helpful comments and agree that our original Discussion did not sufficiently align the opening paragraph with the stated aims and could be interpreted as implying a mechanistic link between reduced CMJ performance and impaired oxygen delivery. In the revised manuscript, the first two paragraphs of the Discussion have been substantially restructured. We now state explicitly that markers such as CK, self-reported wellness and CMJ performance were included primarily to verify that the eccentric squat protocol induced a meaningful EIMD response, and that the NIRS outcomes were then used to characterise any accompanying changes in local oxygenation:

Discussion

*These markers were included to verify that the eccentric squat protocol induced a meaningful EIMD response, and the NIRS outcomes were then used to characterise any accompanying changes in local oxygenation. We therefore interpret the NIRS findings as describing how oxygen delivery and utilisation behave in a muscle that is known to be damaged, rather than as evidence that impaired oxygen availability directly explains the observed decline in jump performance.*

In doing so, we remove any implication that our data show CMJ reductions are mediated by oxygen delivery and instead acknowledge that CMJ decrements are most likely explained by well-described excitation–contraction coupling and structural disturbances associated with EIMD, with NIRS used here to describe the accompanying oxygenation profile.

**I do not understand how you can state “This highlights the sensitivity of NIRS to detect muscle oxygenation deficits following EIMD depends on both the exercise modality employed and the specific measurement and data processing techniques utilised.”. Your findings about the TSI during sprint indicate large effect by for only two variables, and do not reflect a clear impairment. Unless I missed some information, I suggest the author to rephrase this sentence to comply with true findings**.

We agree that our previous wording overstated the implications of our findings and could be interpreted as implying that NIRS clearly detects muscle oxygenation “deficits” after EIMD. In line with the reviewer’s suggestion, we have rephrased this sentence to remove the strong “sensitivity” language and to align more closely with the actual pattern of results (large effect sizes for only a small number of variables and no statistically significant group × time interactions). The revised sentence now reads:

Discussion

*Taken together, these comparisons suggest that the extent to which EIMD-related changes in muscle oxygenation are detectable with NIRS is likely to depend on both the exercise modality and the specific measurement and data processing techniques used, and that brief, field-compatible protocols may capture some, but not all, of the perturbations observed in more intensive or occlusion-based paradigms.*
