# Supplementary material for: Assessment of muscle oxygenation following eccentric exercise-induced muscle damage using near-infrared spectroscopy
Source: PLoS One. 2026 Feb 2;21(2):e0341963. doi: 10.1371/journal.pone.0341963 (PMC12863531; doi:10.1371/journal.pone.0341963)
Supplement: Attachment — Submitted filename: Response to Reviewers Jan2026.docx [file pone.0341963.s004.docx]

**Reviewer #2**

**L 301: Considering the answer provided to reviewer #1, authors mentioned that power output was provided to characterize exercise performance level of this population. The use of the mean power as a reflect of the whole performance during sprint 1 remains however of modest information to translate exercise capacity and the presence of residual fatigue. Did the authors attempt to analyze data about total work provided during sprint (i.e. integral from the torque-time signal)? This element should be more suitable to detect the presence of residual fatigue and performance decrement than the mean value calculated throughout the sprint.**

Thank you for this helpful suggestion. We agree that Sprint 1 mean power alone provides limited insight into exercise capacity and the presence of residual fatigue during a repeated sprint bout. In the revised manuscript, cycle sprint performance is now characterised using both Sprint 1 outputs (mean and peak power) and additional metrics derived from the full 10-sprint set. Specifically, we now include total work across the set (kJ), calculated as the sum of sprint work across completed sprints, where sprint work is derived from the recorded 10 Hz power signal as mean power multiplied by the fixed sprint duration (6 s), and we include performance decrement indices calculated from both mean and peak power across the 10 sprints using an established percentage decrement approach (observed summed output relative to an idealised total based on the best sprint value multiplied by the number of completed sprints). These additions are described in the Methods, reported in the Results, and briefly contextualised in the Discussion. Overall, the expanded cycle analyses did not demonstrate clear changes across the recovery period, and the decrement indices showed greater variability than absolute output metrics.

Methods>Repeated Cycle Sprint

*Sprint 1 mean and peak power were retained as single-sprint descriptors of performance in each session. In addition, repeated sprint performance across the 10-sprint set was characterised using summary metrics derived from all sprints. Mechanical work for each sprint was calculated as the time integral of power:*

$Work \left( kJ \right)=\frac{mean power \left( W \right) \times{6s}^{-1}}{1000}$ *(Equation 3)*

*Total work (kJ) was calculated as the sum of sprint work across sprints 1 to 10. To quantify performance decrement across the set, a percentage decrement index was calculated from mean power as the difference between the idealised total (best sprint mean power × number of completed sprints) and the observed total (sum of mean power across sprints), expressed relative to the idealised total (35). The same decrement approach was also applied to peak power values to provide a complementary index of repeated sprint fatigue.*

Results

*Reliability statistics for repeated cycle sprint performance outcomes are presented in Table 4. Single sprint (Sprint 1 only) outputs, namely Sprint 1 mean power and Sprint 1 peak power, showed high reliability (ICC = 0.96 for both) with low to moderate variability (CV = 6.3% and 5.3%, respectively). Variables derived from all 10 sprints, including total work and best sprint mean power, also demonstrated high repeatability (ICC = 0.97 for both) with low variability (CV = 4.1% to 4.7%). In contrast, fatigue-related indices showed poorer reproducibility, with mean power decrement demonstrating low reliability (ICC = 0.29) and high variability (CV = 42.5%), while peak power decrement showed moderate reliability (ICC = 0.70) but similarly high variability (CV = 39.6%), indicating these decrement measures fluctuate substantially between assessments.*

*…*

*For repeated cycle sprint performance (Table 6), Sprint 1 mean power and Sprint 1 peak power are shown in Figure 2. Across all cycle sprint metrics, changes from baseline displayed 90% confidence intervals that overlapped zero, therefore, standardised effect sizes were considered unclear. Accordingly, there were no significant within-group changes or between-group differences for any cycling sprint outcome (P > 0.05).*

*New Table 4 added*

*Table 4. Summary of reliability statistics of repeated cycle performance obtained from control participants during six repeated assessments.*

|  | Mean ± SD | | | | CV [95% CI] | | TE  [95% CI] | | SWC  [95% CI] | | ICC  [95% CI] | |
| --- | --- | --- | --- | --- | --- | --- | --- | --- | --- | --- | --- | --- |
| *Single sprint (Sprint 1 only)* | | | | | | | | | | | | |
| Sprint 1 mean power (W) | 709.0 | ± | 104.2 | | 6.3 [3.5; 9.0] | | 31.5  [23.7; 49.7] | | 20.8  [12.5; 59.9] | | 0.96  [0.86; 0.99] |  |
| Sprint 1 peak power (W) | 956.4 | ± | 117.2 | 5.3 [2.1; 8.6] | | 43.0  [32.4; 67.8] | | 23.5  [14.1; 67.4] | | 0.96  [0.86; 0.99] | |  |
| *Derived from all 10 sprints* | | | | | | | | | | | | |
| Total work (kJ) | 40.2 | ± | 4.5 | | 4.1 [2.5; 5.8] | | 1.8  [1.4; 2.5] | | 1.0  [0.6; 2.8] | | 0.97  [0.90; 0.99] | |
| Best sprint mean power (W) | 736.4 | ± | 87.8 | | 4.7 [3.3; 6.5] | | 29.3  [22.9; 40.7] | | 17.0  [10.2; 48.8] | | 0.97  [0.91; 0.99] | |
| Mean power decrement (%) | 8.5 | ± | 2.3 | | 42.5  [22.6; 67.6] | | 4.0  [3.2; 5.6] | | 0.4  [0.2; 1.0] | | 0.29  [-1.08; 0.90] | |
| Peak power decrement (%) | 8.2 | ± | 3.8 | | 39.6  [30.8; 48.7] | | 3.4  [2.4; 4.2] | | 0.7  [0.4; 2.0] | | 0.70  [-0.15; 0.88] | |
| Abbreviations: SD, standard deviation; CV, coefficient of variation; TE, typical error; SWC, smallest worthwhile change; ICC, intraclass correlation coefficient; 95% CI, upper and lower bound 95% confidence interval | | | | | | | | | | | | |

*Table 6 amended*

|  | | **Baseline** | | | | **∆ Post 1 h** | | | | **∆ Post 24 h** | | | | **∆ Post 48 h** | | | | **∆ Post 72 h** | | | | **∆ Post 96 h** | | | | |
| --- | --- | --- | --- | --- | --- | --- | --- | --- | --- | --- | --- | --- | --- | --- | --- | --- | --- | --- | --- | --- | --- | --- | --- | --- | --- | --- |
| Mid-thigh girth (cm) | 54.2 | | ± | 4.2 | -0.1 | | ± | 0.5 | 0.2 | | ± | 0.5 | 0.1 | | ± | 0.7 | 0.3 | | ± | 0.6 | -0.1 | | ± | 0.6 |  |  |
| Wellness score | 18.1 | | ± | 1.9 | -2.3 ^L^ | | ± | 2.4 | -1.25 | | ± | 2.7 | -1.8^VL^ | | ± | 3.0 | -1.3 | | ± | 3.4 | 0.1 | | ± | 2.7 |  |  |
| CMJ (cm) | 34.9 | | ± | 6.6 | -3.5^S^ | | ± | 3.9 | -3.71^M^ | | ± | 4.9 | -4.0^M^ | | ± | 6.3 | -3.1^S^ | | ± | 5.5 | -1.4 | | ± | 3.9 |  |  |
| CK (IU) | 146.3 | | ± | 25.3 | 150.9^EL^ | | ± | 107.1 | 1884.0 ^EL^ | | ± | 1430.0 | 1364.6 ^L^ | | ± | 1797.6 | 846.1^M^ | | ± | 1296.4 | 583.6^M^ | | ± | 963.7 |  |  |
| *Single sprint (Sprint 1 only)* | | | | | | | | | | |  |  |  | |  |  |  | |  |  |  | |  |  |  |  |
| Sprint 1 mean power (W) | 666.8 | | ± | 100.0 | -35.9 | | ± | 109.9 | -49.56 | | ± | 114.5 | -55.3 | | ± | 108.4 | -4.78 | | ± | 65.0 | 20.5 | | ± | 65.9 |  |  |
| Sprint 1 peak power (W) | 953.1 | | ± | 144.9 | -52.2 | | ± | 154.4 | -55.8 | | ± | 151.4 | -58.0 | | ± | 158.1 | 4.7 | | ± | 92.8 | 23.0 | | ± | 86.1 |  |  |
| *Derived from all 10 sprints* | | | | | | |  |  |  | |  |  |  | |  |  |  | |  |  |  | |  |  |  |  |
| Total work (kJ) | 38.3 | | ± | 5.3 | -2.0 | | ± | 4.5 | -2.3 | | ± | 5.3 | -2.4 | | ± | 5.8 | -0.4 | | ± | 3.3 | 0.3 | | ± | 3.0 |  |  |
| Best sprint mean power (W) | 691.1 | | ± | 94.0 | -32.8 | | ± | 82.9 | -33.3 | | ± | 87.4 | -49.6 | | ± | 98.6 | -9.5 | | ± | 51.8 | 10.6 | | ± | 53.9 |  |  |
| Mean power decrement (%) | 7.6 | | ± | 4.5 | 0.6 | | ± | 3.4 | 1.3 | | ± | 4.0 | -0.8 | | ± | 3.1 | -0.1 | | ± | 2.5 | 0.8 | | ± | 3.2 |  |  |
| Peak power decrement (%) | 9.0 | | ± | 7.8 | -0.5 | | ± | 5.4 | -0.5 | | ± | 5.7 | -1.3 | | ± | 5.5 | -2.4 | | ± | 5.5 | -1.0 | | ± | 6.7 |  |  |
| Abbreviations: CMJ, countermovement jump; CK, creatine kinase. ∆ = difference between timepoint minus baseline. ^S, M, L, VL, EL^ indicate small, moderate, large, very large or extremely large effect sizes, respectively. | | | | | | | | | | | | | | | | | | | | | | | | | |  |

*Discussion*

*Cycle sprint performance was included to characterise the exercise performance capacity of the cohort and to provide a practical functional context for interpreting the NIRS responses. In the present study, neither Sprint 1 outputs nor the set level cycle metrics demonstrated clear changes across the recovery period, with changes from baseline characterised by confidence intervals overlapping zero and no evidence of between-group differences. Reliability analyses indicated that absolute output metrics, including Sprint 1 mean and peak power, total work, and best sprint mean power, were highly reproducible, whereas decrement indices were notably more variable between repeated assessments. Collectively, these findings suggest that the eccentric squat protocol elicited perceptual, biochemical and jump-based consequences of muscle damage, yet did not translate into a detectable impairment in repeated sprint mechanical output within this brief cycling task, and that fatigue indices derived from repeated sprints may be less stable markers of change than absolute power or work in this context.*

**In the discussion section (no line reference available), there is a mention that a small but consistent upward shift in TSI was observed compared to baseline. This element seems to be determined by the small effect size reported in table 6, but not supported by statistics. Did the authors also consider the smallest worthwhile change for this parameter to support their assumption?**

Thank you for the comment. We agree that this statement requires clear linkage to our pre-specified decision criteria for meaningful change. In addition to null hypothesis testing, we evaluated changes using the larger of the typical error (TE) or the smallest worthwhile change (SWC) derived from the control group, together with 90% confidence intervals for the change scores. For resting TSI, TE exceeded SWC (1.6%TSI vs 0.7%TSI), therefore, TE was used as the conservative threshold to distinguish meaningful change from measurement noise. The upward shift in resting TSI at 24 hours was interpreted as small but clear because the mean change exceeded TE and the 90% confidence interval for the change excluded zero, consistent with the criteria described in the Statistical Analyses. We have revised the Results and Discussion to make explicit that this inference is based on the TE or SWC framework, rather than on null hypothesis significance alone.

Results

*Time-course comparisons of resting TSI, sprint, recovery, phase duration, rate of change and half-recovery time (Table 7) showed no significant main effects or interactions (P > 0.05), implying that, from a conventional null hypothesis perspective, these parameters remained stable across time points and between groups. However, when changes from baseline were evaluated against the pre-specified magnitude-based criteria, several outcomes showed clear deviations. Specifically, Resting TSI at 24 h post-EIMD was interpreted as a small but clear increase because the mean change exceeded the TE derived from control participants (TE 1.6 TSI%, which is larger than the SWC of 0.7 TSI%) and the 90% CI for the change excluded zero (Figure 3). In addition, both recovery after Sprint 1 (∆TSI%) and rate of change (%•s-1) at 1 h post-EIMD demonstrated large standardised effects (Figures 4 & 5) consistent with changes that exceeded the TE thresholds. Collectively, these observations highlight potentially meaningful alterations that are not captured by p-values alone. From a practical standpoint, the presence of large effect sizes, despite nonsignificant ANOVA results, emphasises the value of supplementing null hypothesis tests with magnitude-based interpretations, particularly when dealing with smaller samples or high inter-individual variability.*

*Discussion*

*Our NIRS data demonstrate that resting TSI is highly reproducible, with low CV and excellent ICCs, and that it showed a small but consistent upward shift (~2%) at 24 hours post EIMD compared with baseline. While this pattern was not supported by conventional null hypothesis testing, the mean change exceeded the conservative TE threshold derived from control participants (which was larger than the SWC) and the 90% confidence interval for the change excluded zero, supporting interpretation as a small but clear change rather than measurement noise.*

**Typing error page 80: field compatable assessments : field compatible assessments?**

Thank you for noting this. We have checked the manuscript carefully and could not locate any instances of “field compatable”. In all occurrences, the term is already spelt correctly as “field-compatible assessments”. We have nonetheless rechecked the relevant section to ensure the spelling is consistent throughout the submitted version.

**The conclusion that “EIMD-related changes in muscle oxygenation are detectable with NIRS is likely to depend on both the exercise modality and the specific measurement and data processing techniques used” is not convincing, are rather surprising. Particularly, I am not convinced that “specific measurements and data processing” represent a valid hypotheses to present about physiological responses to eccentric exercise. In my opinion, authors should better rephrase this sentence to provide a clear explanations about whether or not EIMD could be detected and monitored following exercise.**

Thank you for this important point. We agree that our original wording could be interpreted as implying that measurement or data processing alters the underlying physiological response to eccentric exercise, which was not our intent. Our aim was to convey a narrower methodological point, namely that the detectability of EIMD related alterations with NIRS depends on the exercise stimulus and the task used to probe oxygen delivery and utilisation, and that differences in how NIRS variables are extracted can influence signal to noise and therefore sensitivity. We have rephrased the sentence to avoid presenting “data processing” as a physiological hypothesis, and to provide a clearer statement of what our findings do and do not support regarding whether EIMD can be detected and monitored with NIRS.

Discussion

*Taken together, these comparisons indicate that EIMD-related alterations in muscle oxygenation can be detected with NIRS, but the magnitude and timing of detectable changes depend on the damaging stimulus and the exercise task used to interrogate oxygen delivery and utilisation. In the present study, a brief, field-compatible approach identified small to large changes in selected indices at specific time points after EIMD, whereas several other NIRS variables showed no clear change. This suggests that short practical assessments may capture some perturbations in local oxygenation after eccentric exercise, but may be less sensitive than protocols that use a more metabolically demanding exercise task, or that incorporate occlusion based measures.*
